# A Web-Based Training Resource for Therapists to Deliver an Evidence-Based Exercise Program for Rheumatoid Arthritis of the Hand (iSARAH): Design, Development, and Usability Testing

**DOI:** 10.2196/jmir.8424

**Published:** 2017-12-13

**Authors:** Cynthia Swarnalatha Srikesavan, Esther Williamson, Lucy Eldridge, Peter Heine, Jo Adams, Tim Cranston, Sarah E Lamb

**Affiliations:** ^1^ Centre for Rehabilitation Research in Oxford, Botnar Research Centre Nuffield Department of Orthopaedics, Rheumatology and Musculoskeletal Sciences University of Oxford Oxford United Kingdom; ^2^ Oxford Clinical Trials Research Unit, Botnar Research Centre Nuffield Department of Orthopaedics, Rheumatology and Musculoskeletal Sciences University of Oxford Oxford United Kingdom; ^3^ Centre for Innovation and Leadership in Health Sciences University of Southampton Southampton United Kingdom

**Keywords:** hand function, hand exercises, rheumatoid arthritis, online training, implementation

## Abstract

**Background:**

The Strengthening and Stretching for Rheumatoid Arthritis of the Hand (SARAH) is a tailored, progressive exercise program for people having difficulties with wrist and hand function due to rheumatoid arthritis (RA). The program was evaluated in a large-scale clinical trial and was found to improve hand function, was safe to deliver, and was cost-effective. These findings led to the SARAH program being recommended in the UK National Institute for Health and Care Excellence guidelines for the management of adults with RA. To facilitate the uptake of this evidence-based program by clinicians, we proposed a Web-based training program for SARAH (iSARAH) to educate and train physiotherapists and occupational therapists on delivering the SARAH program in their practice. The overall iSARAH implementation project was guided by the 5 phases of the analysis, design, development, implementation, and evaluation (ADDIE) system design model.

**Objective:**

The objective of our study was to conduct the first 3 phases of the model in the development of the iSARAH project.

**Methods:**

Following publication of the trial, the SARAH program materials were made available to therapists to download from the trial website for use in clinical practice. A total of 35 therapists who downloaded these materials completed an online survey to provide feedback on practice trends in prescribing hand exercises for people with RA, perceived barriers and facilitators to using the SARAH program in clinical practice, and their preferences for the content and Web features of iSARAH. The development and design of iSARAH were further guided by a team of multidisciplinary health professionals (n=17) who took part in a half-day development meeting. We developed the preliminary version of iSARAH and tested it among therapists (n=10) to identify and rectify usability issues and to produce the final version.

**Results:**

The major recommendations made by therapists and the multidisciplinary team were having a simple Web design and layout, clear exercise pictures and videos, and compatibility of iSARAH on various browsers and devices. We rectified all usability issues in the preliminary version to develop the final version of iSARAH, which included 4 short modules and additional sections on self-assessment, frequently asked questions, and a resource library.

**Conclusions:**

The use of the ADDIE design model and engagement of end users in the development and evaluation phases have rendered iSARAH a convenient, easy-to-use, and effective Web-based learning resource for therapists on how to deliver the SARAH program. There is also huge potential for adapting iSARAH across different cultures and languages, thus opening more opportunities for wider uptake and application of the SARAH program into practice.

## Introduction

Rheumatoid arthritis (RA) is a chronic inflammatory joint disease that presents with pain, inflammation, stiffness, and reduced muscle strength, joint movements, and joint function [1,2].

Joints of the hands and wrists are very commonly affected in people with RA [2,3], resulting in reduced functional ability of the hands [4-7]. The Strengthening and Stretching for Rheumatoid Arthritis of the Hand (SARAH) program is an individually tailored, progressive exercise program for people with pain and hand function problems due to RA [8,9]. It includes mobility exercises for the hand, wrist, and shoulder and strengthening exercises for the hand and wrist muscles. The exercises are delivered by a therapist with behavioral support strategies for exercise adherence, such as exercise diaries, goal setting, action planning, confidence building, and problem solving, along with routine advice on joint protection, assistive devices, and splints. Between 2009 and 2011, a large, pragmatic, multicenter randomized controlled trial (ISRCTN 89936343) evaluated the SARAH program across 17 National Health Service (NHS) hospitals in the United Kingdom [10]. A total of 490 adults with diagnosed RA, and who had been on a stable drug regimen for at least 3 months, were randomly assigned to receive best practice usual care either alone or in conjunction with the SARAH program. Significant improvements in overall hand function and self-efficacy were seen at 4 and 12 months in participants who received the SARAH program. The program was also found to be safe and cost-effective [10]. Based on this research, the exercise program is now recommended in the UK National Institute for Health and Care Excellence (NICE) guidelines for patients with RA affecting their hands [11].

Due to the success of the program and the NICE recommendations, we are now aiming to disseminate the evidence-based SARAH program to facilitate its use in clinical practice. In the original clinical trial, therapists attended a face-to-face training session (one-half to 1 day in duration) to learn how to deliver the SARAH program. Following the publication of the SARAH clinical trial results, all the patient and therapist materials required to deliver the SARAH program were made available for health care professionals worldwide downloadable from the Oxford Clinical Trials Research Unit (OCTRU) website [12].

However, we recognized the need for a knowledge dissemination tool with the potential to facilitate wider and systematic uptake of the SARAH program by physiotherapists and occupational therapists and its implementation in clinical practice. We, therefore, proposed a free Web-based training program for SARAH, iSARAH [13], to serve this purpose. Web-based training programs use modern telecommunication and information technologies to deliver information and have the capacity to accommodate multimodal learning formats (eg, written materials, multimedia, animations, feedback, and assessments) [14,15]. They can reach many people at their convenience, can overcome geographical barriers, and are cost-effective in terms of time, effort, and travel [15]. Web-based training has the potential to be an effective method of reaching and training health professionals globally [16-20].

The iSARAH implementation project is based on the analysis, design, development, implementation, and evaluation (ADDIE) model, one of the common instructional system design models used for constructing Web-based programs [21-24].

The analysis stage comprises defining the problem, identifying the target knowledge users, and looking for possible solutions to bridge the knowledge-action gap and user-specific needs for the dissemination tool. In the context of the SARAH program, the knowledge-action gap is the evidence-based SARAH program (current knowledge) and its application in practice (action). The targeted users are the physiotherapists and occupational therapists who routinely treat and prescribe hand exercises to people with RA. We proposed to bridge the knowledge-action gap by educating and training the therapists on the SARAH program with a knowledge dissemination tool (iSARAH).

The design stage consists of finding ways to organize and present the content, identifying modes of delivery, and developing an evaluation plan of the dissemination tool. This stage involves conceptualizing and adapting the SARAH program to fit the Web-based iSARAH.

The development stage involves building iSARAH, evaluating its usability issues, and refining iSARAH to develop the final version.

The implementation stage involves making iSARAH available for NHS therapists.

The evaluation stage will include evaluation of learning outcomes such as knowledge, attitudes, intention to implement and user satisfaction with iSARAH, and evaluation of actual use of the SARAH program by iSARAH-trained therapists in real-world settings.

Here we describe the first 3 phases of the iSARAH implementation project.

## Methods

### Phase 1: iSARAH Needs Analysis

Specific objectives of this phase were (1) to explore routine exercise prescription practices and outcomes use among therapists who treat people with RA affecting the hands and wrists, (2) to identify barriers and facilitators to implementing the SARAH program, and (3) to collect therapists’ opinions and preferences on the design, content, and features of iSARAH.

A convenience sample of physiotherapists and occupational therapists of different countries who downloaded the SARAH program materials from the OCTRU website and gave permission to be contacted by the SARAH team were considered eligible for participation in the SARAH survey. Willingness to provide consent for taking part in the survey was the other inclusion criterion.

We developed a survey questionnaire (Multimedia Appendix 1) that focused on routine therapist practice patterns in prescribing hand exercises for people with RA, and their experiences of using the SARAH program in clinical practice since they downloaded the SARAH program materials. We also asked therapists about barriers and enablers to using the SARAH program, and their preferences for the content, design, and structure of iSARAH. We sent invitation emails with a weblink containing information about the survey, along with a consent form and some questions relating to the therapists’ professional background and experience. Access to the survey was allowed for those therapists who provided online consent. Those who consented were asked to complete the survey within 2 weeks. For nonresponders, a reminder email was sent after 2 weeks, followed by a final reminder a week later.

The survey protocol was reviewed and approved by the medical sciences Inter-Divisional Research Ethics Committee at the University of Oxford, Oxford, UK (reference number R43362/RE001). The SARAH survey was developed using LimeSurvey (LimeSurvey GmbH), an open source survey tool, and was hosted by OCTRU, University of Oxford.

### Phase 2: iSARAH Design

Specific objectives of this phase were (1) to design a paper prototype of iSARAH, and (2) to gain feedback from a multidisciplinary group of health professionals and to agree on the content, delivery methods, frequently asked questions (FAQs), and the navigation, layout, and visual appeal features of iSARAH.

The SARAH research team and information technology experts mapped the SARAH program from the SARAH clinical trial to a 3- to 4-hour Web-based training package for therapists and designed a paper prototype. We proposed a half-day meeting with rheumatology clinicians, researchers, and technology experts based on their convenience and availability to attend the meeting. The purpose of this meeting was to gain collective feedback on the prototype and the survey findings to finalize the design of iSARAH. The paper prototype was presented at a half-day multidisciplinary team meeting (n=17) involving a rheumatologist (n=1), occupational therapists and physiotherapists (n=10; 7 of whom were part of the SARAH trial), SARAH trial researchers (n=4), and information technology experts (n=2).

### Phase 3: iSARAH Development and Usability Testing

Specific objectives of this phase were (1) to develop the iSARAH website, (2) to gain end user feedback on the usability, usefulness, ease of use, and confidence in using iSARAH, and (3) to rectify usability issues and further refine iSARAH prior to its implementation.

This phase involved building iSARAH (preliminary version) and evaluating its usability, usefulness, and ease of use and user confidence [25,26]. The usability evaluation protocol was reviewed and approved by the medical sciences Inter-Divisional Research Ethics Committee, University of Oxford (reference number R47560/RE001).

NHS hand therapists (physiotherapists and occupational therapists) who were treating people with RA and lived within 2 hours of travel to Oxford were considered eligible for participation in the usability testing. Willingness to provide signed consent was the inclusion criterion. We invited volunteers via the Centre for Rehabilitation Research in Oxford Twitter page and the online community forum of the Chartered Society of Physiotherapy.

Based on the available evidence that 80% of usability issues can be identified by testing with 5 participants and that 95% can be identified with 9 participants [27,28], we proposed to include 10 therapists who fulfilled the inclusion criteria.

We coordinated individual appointments to attend usability sessions through telephone calls and conducted the sessions at the Botnar Research Centre, University of Oxford. Before evaluation, participants provided signed consent and completed a series of demographic questions. The usability testing procedure was then explained emphasizing that the session was about evaluating iSARAH and not the user. Each session took approximately 90 minutes. The usability testing involved the following procedures.

#### Think-Aloud Procedure

The procedure was facilitated by 1 of the members of the SARAH implementation team. Participants were asked to log on to the iSARAH website by registering with test usernames and passwords. They were then asked to navigate through the website, starting from the home page. They were simultaneously encouraged to talk about what they felt, saw, or thought while browsing. The facilitator observed and took notes as participants were asked to verbalize their thoughts. When participants had difficulties in verbalizing, they were encouraged by a “keep talking” signboard and were minimally assisted with prompts (only when required) by the facilitator. All think-aloud sessions were audio recorded.

#### Self-Reported Questionnaires

We used the Computer System Usability Questionnaire (CSUQ) [29] to evaluate user satisfaction, ease of use, information, and interface of the program on a 7-point Likert scale (1=strongly disagree to 7=strongly agree).

We measured iSARAH usefulness on a 5-point Likert scale (1=not at all useful, 2=slightly useful, 3=moderately useful, 4=very useful, and 5=extremely useful).

We measured overall ease of use on a 5-point Likert scale (1=very difficult, 2=somewhat difficult, 3=neither difficult nor easy, 4=somewhat easy, and 5=very easy).

We measured confidence in using iSARAH on a 5-point Likert scale (1=not at all confident, 2=somewhat confident, 3=not sure, 4=confident, and 5=very confident).

#### Interviews

Using a semistructured interview guide, we asked participants about their experiences in navigating iSARAH. Interviews were conducted for approximately 10 to 15 minutes and were audio recorded. We summarized users’ comments on iSARAH by listening to the audio files and cross-checking a second time.

## Results

**Figure 1:**
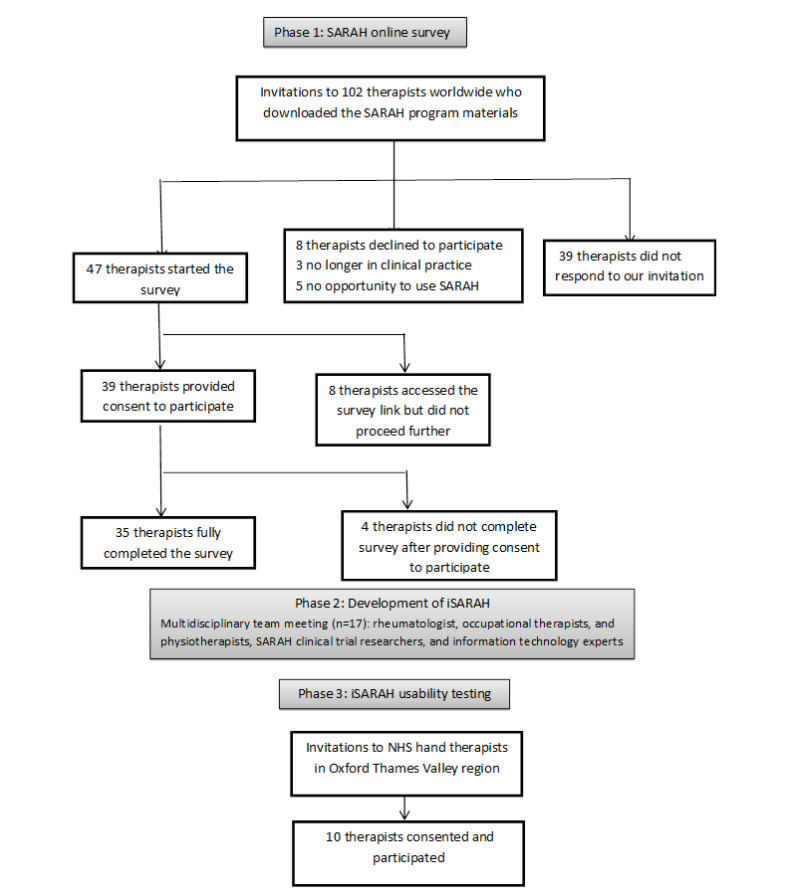
Study flow diagram. SARAH: Strengthening and Stretching for Rheumatoid Arthritis of the Hand; iSARAH: Web-based training program for SARAH; NHS: National Health Service.

Figure 1 shows the flow of participants through the 3 phases of iSARAH.

### Phase 1: iSARAH Needs Analysis

We sent SARAH survey invitations to a total of 102 physiotherapists and occupational therapists who had downloaded the SARAH program materials. Figure 1 displays the flow of the survey participants. Table 1 shows the demographic characteristics of those who took part in the SARAH survey.

**Table 1:** Demographic characteristics of therapists participating in phase 1 and phase 3 of the study.

Demographics		Phase 1 (SARAH^a^ survey) (n=35)	Phase 3 (iSARAH^b^ usability testing) (n=10)
**Age groups in years, n (%)**			
	21-30	4 (11)	0
	30-40	5 (14)	2 (20)
	40-50	16 (46)	5 (50)
	>50	10 (29)	3 (30)
**Sex, n (%)**			
	Male	4 (11)	1 (10)
	Female	31 (89)	9 (90)
**Profession, n (%)**			
	Occupational therapists	18 (51)	7 (70)
	Physiotherapists	17 (49)	3 (30)
**Employment, n (%)**			
	Full-time	18 (51)	6 (60)
	Part-time	17 (49)	4 (40)
**Work setting, n (%)**			
	Public (eg, NHS^c^ hospital)	32 (91)	10 (100)
	Private practice	1 (3)	0
	Other (eg, teaching)	2 (6)	0
**Work experience in years**			
	<5	5 (14)	
	5-10	14 (40)	
	10-15	3 (9)	
	>15	13 (37)	
	Median (IQR^d^)	N/A^e^	17 (9.25)
Hours on Internet/day, Median (IQR)		N/A	2 (0.5)

^a^SARAH: Strengthening and Stretching for Rheumatoid Arthritis of the Hand.

^b^iSARAH: Web-based training program for SARAH.

^c^NHS: National Health Service.

^d^IQR: interquartile range.

^e^N/A: not applicable.

Table 2 describes the key features of the therapists’ current clinical practice. Most respondents saw more than 10 patients per month with RA.

Pain, self-reported hand function, joint range of motion, stiffness, grip and pinch strength, and joint deformities were more commonly evaluated as part of their current practice. Performance-based hand function, 28-joint Disease Activity Score, and activities of daily living were the least evaluated outcomes.

The most common type of exercise prescribed by therapists was active range of motion exercises. Strengthening exercises were also frequently used, as were tendon gliding exercises. Nerve gliding, passive, and isometric exercises were much less commonly prescribed.

Self-management strategies, joint protection, and splinting were more commonly prescribed than thermotherapy, therapeutic gloves, work support, advice on activities of daily living, and electrotherapy.

On average, therapists had 4 sessions with their patients (mean 4, SD 3.9). The frequency of review sessions was mostly either once every 15 days, reported by 11 therapists, or every 1 to 2 months, reported by 9 therapists.

Most therapists used exercise sheets and review appointments to encourage adherence with home exercise programs. Exercise diaries, exercise contracts, and telephone reminders were less commonly used.

**Table 2:** Current practices in therapy management of rheumatoid arthritis affecting the hands (n=35).

Survey items		Therapists n (%)
**Average number of rheumatoid arthritis patients seen per month**		
	>15	12 (34)
	10-15	11 (31)
	5-10	3 (9)
	1-5	9 (26)
**Hand outcomes evaluated**		
	Pain	33 (94)
	Self-reported hand function	28 (80)
	Joint range of motion	26 (74)
	Stiffness	26 (74)
	Grip and pinch strength	22 (63)
	Joint deformities	19 (54)
	Performance-based hand function	13 (37)
	Disease Activity Score 28 and activities of daily living	4 (11)
**Types of hand exercises prescribed**		
	Active range of motion	34 (97)
	Strength	27 (77)
	Tendon gliding	20 (57)
	Nerve gliding, passive, or isometric	4 (11)
**Other treatments**		
	Self-management and coping strategies	32 (91)
	Joint protection advice	30 (86)
	Splinting	24 (69)
	Thermotherapy	15 (43)
	Therapeutic gloves, work support, and advice on activities of daily living	6 (17)
	Electrotherapy	2 (6)
**Methods to encourage exercise adherence**		
	Exercise sheets	33 (94)
	Review appointments	29 (83)
	Exercise diaries	8 (23)
	Exercise contracts	2 (6)
	Telephone reminders	1 (3)
**SARAH**^a^ **program prescribed in practice**		
	Yes	26 (74)
	No	9 (26)

^a^SARAH: Strengthening and Stretching for Rheumatoid Arthritis of the Hand.

About 74% (n=26) of the therapists delivered the SARAH program in their clinical practice, and on average had prescribed the program to 17 (SD 22) of their patients since downloading the materials. More than 50% (17/26) of the therapists who delivered the SARAH program did not find any aspect of SARAH that made it difficult to put into practice. They reported that the SARAH therapist manual, the exercise sheets with photographs, and the strong evidence base facilitated their use of the SARAH program in their daily practice. Other therapists reported issues with time, funding for exercise equipment, and inability to complete review assessments and exercise contracts.

Therapists who did not use the SARAH program (n=9) reported a lack of appropriate patients to be prescribed the SARAH program, budget, time, and their routine prescription of hand exercises like the SARAH program as main reasons for nonimplementation. Table 3 presents barriers and facilitators identified by therapists who completed the survey (n=35).

We asked therapists what they would like to see in a Web-based training program if one were available. Textbox 1 lists their suggestions.

### Phase 2: iSARAH Design

Following the multidisciplinary team meeting, we identified the specific need to educate and train therapists on the behavioral support strategies and proposed a separate module on this topic. We agreed that a section addressing common questions that might be raised by therapists about the SARAH program in real-world settings should be included in an FAQ section of iSARAH. Attendees provided suggestions for framing these questions. Based on discussions about the iSARAH prototype and SARAH survey findings, the team suggested the following recommendations: (1) to provide weblinks within the text for additional information on a topic, for example, Splints in RA, (2) to provide a progression status bar to enable users to know where they are in the training, (3) to use consistent names for exercises, (4) to have a separate educational video on joint protection advice, (5) to have a separate module on behavioral support strategies, (6) to have SARAH exercises demonstrated through videos and photographs, (7) to have brief modules, (8) to have a plain layout and use optimal font sizes (14 point), (9) to have an official email support to address technical enquiries, and (10) to ensure iSARAH adapts across different types of Internet browsers and computers at NHS settings and other telecommunication devices.

Specific recommendations were also made regarding the behavioral strategies module: (1) to provide examples of general goals relating to upper limb function to aid therapists with goal setting, and (2) to include model scenarios on filling in the personal exercise guide and Barriers and Facilitators form.

**Table 3:** Barriers and facilitators reported by therapists who completed the SARAH^a^ survey (n=35).

Barriers and facilitators	Always a barrier n (%)	Sometimes a barrier n (%)	Neither a barrier nor a facilitator n (%)	Sometimes a facilitator n (%)	Always a facilitator n (%)
Time	7 (20)	16 (46)	11 (31)	1 (3)	0 (0)
Forgetting to use	2 (6)	10 (29)	21 (60)	0 (0)	2 (6)
Belief in its effect on patients	0 (0)	0 (0)	12 (34)	10 (29)	13 (37)
Influence of peers	0 (0)	5 (14)	23 (66)	6 (17)	1 (3)
The need to change practice	0 (0)	5 (14)	14 (40)	8 (23)	8 (23)
Instructions to deliver the program	0 (0)	4 (11)	12 (34)	10 (29)	9 (26)
Current caseload	1 (3)	6 (17)	9 (26)	9 (26)	10 (29)
SARAH exercise equipment	7 (20)	11 (31)	14 (40)	1 (3)	2 (6)
SARAH patient materials	5 (14)	11 (31)	10 (29)	3 (9)	6 (17)

^a^SARAH: Strengthening and Stretching for Rheumatoid Arthritis of the Hand.

**Therapists’ suggestions for what a Web-based training program should have.**

- A simple Web layout and design
- Exercise photographs and videos with clear instructions
- Brief training modules
- A self-assessment section
- Simplified Strengthening and Stretching for Rheumatoid Arthritis of the Hand (SARAH) patient materials
- Instructions on how to complete the personal exercise guide, exercise diary, and Barriers and Facilitators form
- Downloadable SARAH program materials
- Online support for technical queries
- Links for patients to access information or complete outcomes online
- PowerPoint teaching materials
- Compatibility with different browsers, such as Internet Explorer and Google Chrome, and responsiveness in different devices, such as mobiles and tablets

**Recommendations to guide the implementation and evaluation phases of the Web-based training for Strengthening and Stretching for Rheumatoid Arthritis of the Hand (iSARAH).**

1. Make it clear in iSARAH that the SARAH program is flexible and will be feasible to complete at the user’s convenience.
2. Send monthly email reminders to iSARAH-trained therapists.
3. Signpost therapists and their patients to resources needed to deliver the program, which could, ideally, be purchased at a discounted rate (eg, therapeutic putty, resistance bands).
4. Provide multiple hard copies of the SARAH patient materials at no cost to iSARAH-trained therapists for use in clinical practice, if required.
5. Demonstrate high credibility by incorporating information about the SARAH research team and all SARAH peer-reviewed publications.
6. Propose pain and self-reported hand function as the main outcomes for the evaluation phase.

To facilitate effective implementation of the SARAH program by iSARAH-trained therapists in actual practice, we also discussed ways to minimize major implementation barriers reported in the survey (time limitations, forgetting, and difficulties in access to and cost of SARAH exercise equipment and patient materials). Clinicians who had worked on the SARAH clinical trial raised some issues with the original forms used in the trial, and they proposed ways to streamline these forms to make them easier to use.

Textbox 2 lists suggestions to guide the implementation evaluation phases of the SARAH program.

### Phase 3: iSARAH Development and Usability Testing

#### Development: Preliminary iSARAH

iSARAH was built on a Moodle platform (release version 3.1; Moodle Pty Ltd) by the OCTRU information technology team, customized and styled using the Essential Theme add-on. An overview of iSARAH (preliminary version) is provided below.

##### Landing Page

The landing page introduced the iSARAH with a brief statement about the purpose of the website, site contact information, privacy policy, and the modules. Other features included a 2.5-minute preliminary iSARAH promotional video and a prominent widget for logging on to the training.

##### Modules

Module 1 covered clinical aspects of RA, benefits of exercises in RA, UK guidelines in the management of RA, and information about the SARAH clinical trial.

Module 2 covered development and physiological principles of the SARAH program, behavioral support strategies, and instructions on how to deliver the SARAH program.

Module 3 covered the self-assessment.

Module 4 included FAQs to inform the delivery of the SARAH program in different practice settings and patient scenarios.

##### Resource Library

All text materials required to deliver the SARAH program (eg, exercise booklets and videos, exercise diary, RA patient education booklets) and additional reference documents, such as SARAH trial publications, were archived in the resource library.

##### Delivery of Content

A combination of text, photographs, tables, and videos was used to deliver the training. Preliminary videos were produced for iSARAH promotion and instruction purposes of the training.

##### Visual Design and Navigation

A simple Web layout was used consistently across modules to reduce distraction and information overload.

#### iSARAH Usability Testing

Table 1 presents demographics of participants in the usability testing.

##### Think-Aloud Procedure

One of the major usability issues we observed was the difficulty in navigating from the end of one module to the next (eg, from the last page of Module 1 to the first page of Module 2), as there were no direct buttons to take users to the following module. Instead, participants had to click the respective module tabs on the top of the screen to navigate between modules or to proceed to the next module. We also noticed that some additional tabs appearing within the Moodle platform were confusing for the participants.

Hyperlinks to reference documents such as SARAH trial publications and patient materials were reported to be repetitive and distracting. Participants said that photographs showing RA hands and activities of daily living, and other illustrations, did not add to iSARAH but instead occupied screen space and led them to frequently scrolling down to read the whole page. In the self-assessment module, when participants entered an incorrect response to a question, they couldn’t find a feature to signpost to the correct response in the respective module. They also reported that information about the SARAH team on the home page was not adequate.

##### Self-Reported Questionnaires

The CSUQ showed that participants overall found iSARAH simple, easy to use, and easy to understand, and they were satisfied in using it (Table 4). There was an overall agreement that participants could complete their work quickly and efficiently and recover from any unexpected technical mistakes. There was some uncertainty as to whether the system gave error messages and informed them how to fix problems. Results from Likert scales (Table 4) indicated that participants rated iSARAH as useful and easy to use, and that they were confident about using it.

**Table 4:** Questionnaire scores of iSARAH^a^ usability testing (n=10).

Questionnaire		Median (IQR^b^)
**Computer System Usability Questionnaire items on 1-7 scale**^c^		
	Overall, I am satisfied with how easy it is to use this system	6 (0.75)
	It was simple to use this system	6 (0)
	I can effectively complete my work quickly using this system	5 (1.0)
	I am able to complete my work quickly using this system	5 (0)
	I am able to efficiently complete my work using this system	5 (1.0)
	I feel comfortable using this system	6 (1.5)
	It was easy to learn to use this system	6 (0.75)
	I believe I became productive quickly using this system	6 (1.0)
	The system gives error messages that clearly tell me how to fix problems	4 (0)
	Whenever I make a mistake using this system, I recover easily and quickly	5 (1.0)
	The information (such as online help, on-screen messages, and other documentation) provided with this system is clear	6 (1.0)
	It is easy to find the information I needed	6 (2.0)
	The information provided for the system is easy to understand	6 (1.0)
	The information is effective in helping me complete the tasks and scenarios	6 (0.75)
	The organization of information on the system screens is clear	5.5 (1.0)
	The interface of the system is pleasant	6 (1.0)
	I like the using the interface of this system	6 (1.0)
	This system has all the functions and capabilities I expect it to have	6 (0.75)
	Overall, I am satisfied with this system	6 (0)
**Likert scale scores of perceived usefulness, ease of use, and confidence in using iSARAH**		
	Usefulness (1=not at all useful; 5=extremely useful)	4.0 (1)
	Ease of use (1=very difficult; 5=very easy)	4.0 (0)
	Confidence in using iSARAH (1=not at all confident; 5=very confident)	4.5 (1)

^a^iSARAH: Web-based training for Strengthening and Stretching for Rheumatoid Arthritis of the Hand.

^b^IQR: interquartile range.

^c^1=strongly disagree, 2=disagree, 3=somewhat disagree, 4=neither, 5=somewhat agree, 6=agree, 7=strongly agree.

##### Interviews

In general, users found that iSARAH was a detailed and helpful learning resource for therapists. The most common comments were that participants liked the Web layout, tabs for modules, exercise videos, and the whole content. Some key suggestions provided were to create videos of good sound quality, and to remove excess text and photographs to keep the information relevant and clear.

#### Modifications Made to Produce the Final Version of iSARAH

We revised iSARAH to address all major usability issues identified from the think-aloud procedure and interviews (Table 5). We produced good-quality promotional (Multimedia Appendix 2) and instructional videos using media professionals and removed all irrelevant photographs to allow more screen space. We minimized repetitive links to reference documents and patient materials within modules. We set up clear-cut tabs to navigate between the end of a module and the start of subsequent modules. The SARAH implementation team further reviewed the final version of iSARAH (Multimedia Appendix 3) for content, navigation issues, and grammar.

Prior to official launch on April 3, 2017, we tested and activated the following features: (1) online user registration page, (2) online feedback questionnaire on perceived usefulness, satisfaction, and intention to use the SARAH program in future practice, and (3) download option for the training completion certificate.

**Table 5:** Major usability issues identified and rectifications made.

Usability issues	Solutions implemented in the final iSARAH^a^
Navigation between the last and first pages of consecutive modules was difficult.	Navigation was made easy by adding buttons to take the user from the last page of the previous module to the first page of next module.
Different-colored text was hard to follow.	Only 2 colors were used: black for text and blue for weblinks.
Sections A, B, and C of Module 2 were confusing.	Sections A, B, and C of Module 2 were categorized as separate modules: modules 2, 3, and 4.
Having FAQs^b^ and self-assessment labelled as modules was irrelevant.	FAQs and self-assessment were labelled with their same names for more clarity.
Resource library documents were not opening in a separate window, and it was confusing when participants closed the document and wanted to access their last seen page of the training.	Documents were set to easily open up and close in a separate window that will allow users stay on their last seen page of the training.
Too many links within the modules was distracting.	Repetitive links were removed.
Too much scrolling was annoying because of photographs occupying space.	Photographs were removed to allow more space for text and less scrolling.
For the self-assessment, when an incorrect answer was entered, participants were not directed to find correct answers in the respective modules.	The self-assessment section was set to point out incorrect responses. When the user provides an incorrect response, he or she will be directed to the relevant module to learn more on the particular question.
The home page did not cover all essential information about the SARAH^c^ program and SARAH team.	More information on the SARAH program, the SARAH team, and the host organization was added. A promo video was produced.
Some Moodle features (eg, tags, buttons) were distracting.	All irrelevant buttons and tags were removed.
The quality of videos could be improved.	Good-quality videos were produced.
A patient could demonstrate exercises in exercise videos.	Exercise videos with a patient volunteer demonstrating the exercises were produced.
There was too much text to read.	The text was reduced, and more bullet points were used.

^a^iSARAH: Web-based training for Strengthening and Stretching for Rheumatoid Arthritis of the Hand.

^b^FAQs: Frequently Asked Questions.

^c^SARAH: Strengthening and Stretching for Rheumatoid Arthritis of the Hand.

## Discussion

The overall purpose of this paper was to present how we developed a Web-based implementation tool (iSARAH) and produced the final version suitable for implementation. The strength of this work is that it followed a recognized model for the construction of Web-based programs [21-24].

### Principal Findings

Engagement with users through the SARAH survey allowed us to identify current practice and learning needs to ensure iSARAH was fit for purpose. From the survey, we established that the exercises included in the SARAH program were commonly used by therapists [6,7,30,31] but the behavioral change techniques were likely to be less familiar [8-10]. It also gave us insight into potential barriers to implementation. Respondents provided information about the features they would like to see in a Web-based training program, and this directly informed the design of the program. Survey findings also directly influenced the selection of outcomes for the evaluation phase of implementation.

Engagement with users continued during the design phase with a face-to-face meeting, as well as carrying out usability testing. Usability testing was essential to producing a user-friendly website that could be deployed for implementation. We believe this has resulted in a flexible learning experience for users, which is easy to navigate with unlimited access. We included FAQs and self-assessment to ensure that therapists have adequate training and skills to efficiently apply for the SARAH program in actual practice.

The next step is to evaluate the impact of iSARAH training on actual implementation of the SARAH program, including the impact on knowledge and skills of therapists, implementation rates, and patient outcomes. We know from our previous work [32] that training alone may not result in implementation [33]. A Web-based training developed to facilitate the implementation of a cognitive behavior approach for low back pain was shown to be as effective as face-to-face training regarding knowledge and confidence, but actual implementation rates were low and further enhancement of the training was required [33]. We have tried to identify potential barriers to implementation during the development phase of this project so that these are addressed by the Web-based training.

### Limitations

This study has some limitations. First, we neither used observational analysis with video recordings in the think-aloud procedure to observe users’ interactions with iSARAH, nor conducted a systematic qualitative analysis of participants’ interviews. Second, the CSUQ and Likert scales have not been tested for reliability and validity in the target population. Hence, the range of scores should be interpreted with caution. Third, the SARAH survey participants were familiar with the SARAH program and hence their responses were prone to the risk of volunteer bias. Additionally, with a low consent rate (39 of 102 participants, 38.2%), the survey findings are at the risk of nonresponse bias from people who did not participate or respond. Fourth, we did not employ iterative cycles of usability testing—that is, consecutive cycles of testing until the point when no further usability issues were identified—but we used the feedback from all participants in a one-off cycle to refine iSARAH.

Evidence-based therapies have been found to be poorly disseminated into routine practice [34]. Some of the barriers often reported by health professionals in practicing evidence are the lack of access to evidence resources [35-37] and nonavailability of the evidence resources in usable formats [38]. In the context of implementing the evidence-based SARAH program, we believe that the easy and free access for health professionals to the SARAH program in a simplified Web-based format has overcome these barriers. We foresee that the training of qualified health professionals directly involved in the rehabilitation of people with RA of the hands would increase their knowledge of the evidence (the SARAH program), and build their skills and confidence to deliver it in practice. The Web-based training would also be a time-saving learning resource that is also potentially flexible in terms of learning [39] for health professionals from diverse backgrounds of Internet use habits and computer skills. Further, the content of the iSARAH can be adapted [19] for language and cultural differences to assist wider implementation. Thus, it would open opportunities to disseminate the SARAH program among therapists across the world who have limited or no access to the SARAH training.

### Next Steps

In our next steps toward opening more opportunities for wider uptake and application of the SARAH program into clinical practice, there is also a huge potential for adapting iSARAH across different cultures and languages across the world.

### Conclusions

To our knowledge, iSARAH is the first Web-based learning resource for therapists on an evidence-based hand exercise program. A systematic design approach by using the ADDIE model and involving end users has been successful in developing a user-centered iSARAH.

Our ongoing work on the impact evaluation among therapists who completed iSARAH and a service evaluation in people treated by SARAH-trained therapists will provide more insights on the uptake of the SARAH program in actual practice.

## Appendix

Multimedia Appendix 1 SARAH therapist survey questionnaire.

Multimedia Appendix 2 iSARAH promotional video.

Multimedia Appendix 3 iSARAH website screenshots.

## Abbreviations

ADDIE: analysis, design, development, implementation, and evaluation
CSUQ: Computer System Usability Questionnaire
FAQs: frequently asked questions
iSARAH: Web-based training for Strengthening and Stretching for Rheumatoid Arthritis of the Hand
NHS: National Health Service
NICE: National Institute for Health and Care Excellence
OCTRU: Oxford Clinical Trials Research Unit
RA: rheumatoid arthritis
SARAH: Strengthening and Stretching for Rheumatoid Arthritis of the Hand
