# Supplementary material for: A Web-Based Training Resource for Therapists to Deliver an Evidence-Based Exercise Program for Rheumatoid Arthritis of the Hand (iSARAH): Design, Development, and Usability Testing
Source: J Med Internet Res. 2017 Dec 13;19(12):e411. doi: 10.2196/jmir.8424 (PMC5745347; doi:10.2196/jmir.8424)
Supplement: Multimedia Appendix 1 [file jmir_v19i12e411_app1.pdf]

i-SARAH Therapist Survey v1.0

Thank you for participating in our survey. Please select the appropriate responses for the following questions.

There are 22 questions in this survey

There are 27 questions in this survey

Study Information

[]

An online survey of therapists' current practice and use of the SARAH exercise programme

Study Information

This online survey is led by the researchers based at the University of Oxford. You are being invited to participate in this survey because you downloaded the SARAH trial materials from the Oxford Clinical Trials Research Unit (OCTRU) website and gave your permission to be contacted by us.

This survey will help us understand the clinical practices among physiotherapists and occupational therapists in prescribing hand exercises to patients with rheumatoid arthritis. We are also interested in how you have used the SARAH exercise programme materials that you downloaded from the OCTRU website.

It is entirely up to you to participate in this survey and if you decide to take part, you are free to withdraw anytime without giving a reason. If you choose to participate, you will be asked to tick in the 'Yes' box against the consent statement on the next page. You will then have access to a brief online survey questionnaire which will take approximately 7-10 minutes to complete.

You will be given a unique study number and all information provided by you will be kept strictly

confidential. The survey data will be stored in a password protected database system managed by the University of Oxford which will be accessed by the study researchers only. For audit and data monitoring purposes, it may be made accessible to responsible members of the University of Oxford. Research data is retained for 5 years. The study will comply with the Data Protection Act and any personal details held by the study team will be stored separately from any participant study data. Personal details will be destroyed on completion of the study.

There are no benefits or risks to participants taking part in this survey.

This study has been reviewed by, and received ethics clearance through, the University of Oxford Central University Research Ethics Committee (CUREC).

If you need further information about the survey, please contact us at, sarahexercise@ndorms.ox.ac.uk

If you have a concern about any aspect of this project, please speak to Dr Esther Williamson (01865 223 120) who will do her best to answer your query. The researcher should acknowledge your concern within 10 working days and give you an indication of how she intends to deal with it. If you remain unhappy or wish to make a formal complaint, please contact the chair of the ***Medical Sciences Inter-Divisional Research Ethics Committee*** *at the University of Oxford (Email: ethics@medsci.ox.ac.uk; Address: Research Services, University of Oxford, Wellington Square, Oxford OX1 2JD) who will seek to resolve the matter in a reasonably expeditious manner.*

Thank you for taking your time to read this information sheet.

Dr. Esther Williamson

esther.williamson@ndorms.ox.ac.uk

On behalf of the SARAH implementation study team

The Centre for Rehabilitation Research

Nuffield Department of Orthopaedics, Rheumatology and Musculoskeletal Sciences (NDORMS) University of Oxford

Postal address: The Botnar Research Centre, Old Road, Headington, Oxford OX3 7LD

Consent

[]I confirm that I have read the study information. I understand that my participation is purely voluntary and that I am free to withdraw at any time without giving a reason. *

Please choose **only one** of the following:

- ○ Yes
- ○ No

PostConsent

[]Thank you for agreeing to participate in our survey. Please proceed to the survey.

Only answer this question if the following conditions are met:
Answer was 'Yes' at question '2 [consent]' (I confirm that I have read the study information. I understand that my participation is purely voluntary and that I am free to withdraw at any time without giving a reason.)

[]We thank you for your interest in our survey

Only answer this question if the following conditions are met:
Answer was 'No' at question '2 [consent]' (I confirm that I have read the study information. I understand that my participation is purely voluntary and that I am free to withdraw at any time without giving a reason.)

Survey Questions

[]1) Gender *

Only answer this question if the following conditions are met:
Answer was 'Yes' at question '2 [consent]' (I confirm that I have read the study information. I understand that my participation is purely voluntary and that I am free to withdraw at any time without giving a reason.)

Choose one of the following answers

Please choose **only one** of the following:

- ◯ Male
- ◯ Female

[]2) Age *

Only answer this question if the following conditions are met:
Answer was 'Yes' at question '2 [consent]' (I confirm that I have read the study information. I understand that my participation is purely voluntary and that I am free to withdraw at any time without giving a reason.)

Choose one of the following answers

Please choose **only one** of the following:

- ◯ 21-30 years
- ◯ 30-40 years
- ◯ 40-50 years
- ◯ >50 years

[]3) Profession *

Only answer this question if the following conditions are met:
Answer was 'Yes' at question '2 [consent]' (I confirm that I have read the study information. I understand that my participation is purely voluntary and that I am free to withdraw at any time without giving a reason.)

Choose one of the following answers

Please choose **only one** of the following:

- ◯ Physiotherapy
- ◯ Occupational therapy

[]4) Professional qualification *

Only answer this question if the following conditions are met:
Answer was 'Yes' at question '2 [consent]' (I confirm that I have read the study information. I understand that my participation is purely voluntary and that I am free to withdraw at any time without giving a reason.)

Choose one of the following answers
If you choose 'Other (please specify)' please also specify your choice in the accompanying text field.

Please choose **only one** of the following:

- ◯ Graduate degree
- ◯ Post-graduate degree
- ◯ Other (please specify) ____________

[]5) Employment *

Only answer this question if the following conditions are met:
Answer was 'Yes' at question '2 [consent]' (I confirm that I have read the study information. I understand that my participation is purely voluntary and that I am free to withdraw at any time without giving a reason.)

Choose one of the following answers

Please choose **only one** of the following:

- Full time
- Part time

[]6) Practice environment *

Only answer this question if the following conditions are met:
Answer was 'Yes' at question '2 [consent]' (I confirm that I have read the study information. I understand that my participation is purely voluntary and that I am free to withdraw at any time without giving a reason.)

Choose one of the following answers
If you choose 'Other:' please also specify your choice in the accompanying text field.

Please choose **only one** of the following:

- Public hospital (e.g. NHS Hospital)
- Private hospital
- Private practice
- Other ________

[]7) Country *

Only answer this question if the following conditions are met:
Answer was 'Yes' at question '2 [consent]' (I confirm that I have read the study information. I understand that my participation is purely voluntary and that I am free to withdraw at any time without giving a reason.)

Choose one of the following answers

Please choose **only one** of the following:

- United Kingdom (England/Wales/Scotland/Northern Ireland)
- Republic of Ireland
- Argentina
- Spain
- United States of America
- Belgium
- Australia
- Sweden
- Iceland
- Canada
- Germany
- Norway
- Netherlands

- Switzerland
- Denmark
- Japan
- Malaysia
- Italy
- New Zealand

[]8) How long have you been treating patients with RA? *

Only answer this question if the following conditions are met:
Answer was 'Yes' at question '2 [consent]' (I confirm that I have read the study information. I understand that my participation is purely voluntary and that I am free to withdraw at any time without giving a reason.)

Choose one of the following answers

Please choose **only one** of the following:

- < 5 years
- 5-10 years
- 10-15 years
- >15 years

[]9) On average, how many patients with RA would you see each month? *

Only answer this question if the following conditions are met:
Answer was 'Yes' at question '2 [consent]' (I confirm that I have read the study information. I understand that my participation is purely voluntary and that I am free to withdraw at any time without giving a reason.)

Choose one of the following answers

Please choose **only one** of the following:

- 1-5
- 5-10
- 10-15
- >15

[]10) Which outcomes do you evaluate in your patients with RA affecting their hands? *

Only answer this question if the following conditions are met:
Answer was 'Yes' at question '2 [consent]' (I confirm that I have read the study information. I understand that my participation is purely voluntary and that I am free to withdraw at any time without giving a reason.)

Check all that apply

Please choose **all** that apply:

- [ ] Pain
- [ ] Stiffness
- [ ] Joint range of motion

- ☐ Grip and pinch strength
- ☐ Self-reported hand function
- ☐ Performance based function
- ☐ Joint deformity
- ☐ Other: ______

[]11) Which types of hand exercises do you prescribe for your patients with RA affecting their hands? *

Only answer this question if the following conditions are met:
Answer was 'Yes' at question '2 [consent]' (I confirm that I have read the study information. I understand that my participation is purely voluntary and that I am free to withdraw at any time without giving a reason.)

Check all that apply

Please choose **all** that apply:

- ☐ Tendon gliding exercises
- ☐ Active range of motion exercises
- ☐ Strengthening exercises
- ☐ Other: ______

[]12) Which other treatments do you use in treating your patients with RA affecting their hands? *

Only answer this question if the following conditions are met:
Answer was 'Yes' at question '2 [consent]' (I confirm that I have read the study information. I understand that my participation is purely voluntary and that I am free to withdraw at any time without giving a reason.)

Check all that apply

Please choose **all** that apply:

- ☐ Electrotherapy modalities
- ☐ Thermotherapy modalities
- ☐ Joint protection advice
- ☐ Self-management/coping strategies
- ☐ Splinting
- ☐ Other: ______

[]13) After the initial assessment session, how many review sessions, <u>on average</u>, would you provide for patients with RA affecting their hands? Please enter you answer in the box. *

Only answer this question if the following conditions are met:
Answer was 'Yes' at question '2 [consent]' (I confirm that I have read the study information. I understand that my participation is purely voluntary and that I am free to withdraw at any time without giving a reason.)

Only numbers may be entered in this field.

Please write your answer here:

[]14) How often would you review your patients with RA affecting their hands? *

Only answer this question if the following conditions are met:
Answer was 'Yes' at question '2 [consent]' (I confirm that I have read the study information. I understand that my participation is purely voluntary and that I am free to withdraw at any time without giving a reason.)

Choose one of the following answers

Please choose **only one** of the following:

- ◯ Once a week
- ◯ Once a fortnight
- ◯ Every 1-2 months
- ◯ Every 2-3 months
- ◯ Every 3-6 months
- ◯ More than 6 months

[]
15) What methods do you use to encourage patients with RA to do their hand exercises?

(Check any that apply) *

Only answer this question if the following conditions are met:
Answer was 'Yes' at question '2 [consent]' (I confirm that I have read the study information. I understand that my participation is purely voluntary and that I am free to withdraw at any time without giving a reason.)

Check all that apply

Please choose **all** that apply:

- ☐ Provide exercise sheets
- ☐ Provide exercise diaries
- ☐ Complete an exercise contract
- ☐ Review at appointments
- ☐ Telephone reminders
- ☐ I don't use any of the above methods
- ☐ Other:

[]16) Have you used the SARAH exercise programme in your clinical practice since you downloaded the SARAH materials? *

Only answer this question if the following conditions are met:
Answer was 'Yes' at question '2 [consent]' (I confirm that I have read the study information. I understand that my participation is purely voluntary and that I am free to withdraw at any time without giving a reason.)

Please choose **only one** of the following:

- ◯ Yes

○ No

[]17) For approximately how many patients with RA have you prescribed the SARAH exercise programme? Please enter your answer in the box.

Only answer this question if the following conditions are met:
Answer was 'Yes' at question '20 [sarahmaterials]' (16) Have you used the SARAH exercise programme in your clinical practice since you downloaded the SARAH materials?) *and* Answer was 'Yes' at question '2 [consent]' (I confirm that I have read the study information. I understand that my participation is purely voluntary and that I am free to withdraw at any time without giving a reason.)

Only an integer value may be entered in this field.

Please write your answer here:

[]18) What did you find most helpful about the SARAH exercise programme when putting it into practice? Please add your comments in the box below. *

Only answer this question if the following conditions are met:
Answer was 'Yes' at question '20 [sarahmaterials]' (16) Have you used the SARAH exercise programme in your clinical practice since you downloaded the SARAH materials?) *and* Answer was 'Yes' at question '2 [consent]' (I confirm that I have read the study information. I understand that my participation is purely voluntary and that I am free to withdraw at any time without giving a reason.)

Please write your answer here:

[]19) Was there any aspect of the SARAH programme that made it difficult to put into practice? *

Only answer this question if the following conditions are met:
Answer was 'Yes' at question '20 [sarahmaterials]' (16) Have you used the SARAH exercise programme in your clinical practice since you downloaded the SARAH materials?) *and* Answer was 'Yes' at question '2 [consent]' (I confirm that I have read the study information. I understand that my participation is purely voluntary and that I am free to withdraw at any time without giving a reason.)

Please choose **only one** of the following:

○ Yes

○ No

[]If yes, please comment in the box below: *

Only answer this question if the following conditions are met:

Answer was 'Yes' at question '23 [sarahnegatives]' (19) Was there any aspect of the SARAH programme that made it difficult to put into practice?) *and* Answer was 'Yes' at question '2 [consent]' (I confirm that I have read the study information. I understand that my participation is purely voluntary and that I am free to withdraw at any time without giving a reason.)

Please write your answer here:

[]20) If you have not prescribed the SARAH exercise programme to any of your RA patients, what was the main reason for this? Please add your comments in the box below *

Only answer this question if the following conditions are met:
Answer was 'No' at question '20 [sarahmaterials]' (16) Have you used the SARAH exercise programme in your clinical practice since you downloaded the SARAH materials?) *and* Answer was 'Yes' at question '2 [consent]' (I confirm that I have read the study information. I understand that my participation is purely voluntary and that I am free to withdraw at any time without giving a reason.)

Please write your answer here:

[]
21) To what extent to the following factors influence your use of the SARAH exercise programme in your daily practice? We have described each factor as being a **barrier** (prevents you using the SARAH exercise programme) or a **facilitator** (help you to use the SARAH exercise programme).

(Please choose one answer for each factor) *

Only answer this question if the following conditions are met:
Answer was 'Yes' at question '2 [consent]' (I confirm that I have read the study information. I understand that my participation is purely voluntary and that I am free to withdraw at any time without giving a reason.)

Please choose the appropriate response for each item:

	Always a barrier	Sometimes a barrier	Neither a barrier nor a facilitator	Sometimes a facilitator	Always a facilitator
Time	○	○	○	○	○
Forgetting to use	○	○	○	○	○
Belief in its effect on patients	○	○	○	○	○
Influence of peers	○	○	○	○	○
The need to change practice	○	○	○	○	○
The instructions describing how to deliver the SARAH exercise programme	○	○	○	○	○
Confidence in prescribing the SARAH exercise programme	○	○	○	○	○
Current caseload	○	○	○	○	○
The need to have the SARAH exercise programme equipment (e.g. resistance band, therapeutic putty)	○	○	○	○	○
The patient materials - exercise instruction sheets, exercise planner and diary	○	○	○	○	○

[]22) We are designing an online SARAH exercise programme training package to help therapists deliver the programme. What would you like to see in this programme (for example, it's structure, visual design, website features)? Please add your comments in the box below *

Only answer this question if the following conditions are met:
Answer was 'Yes' at question '2 [consent]' (I confirm that I have read the study information. I understand that my participation is purely voluntary and that I am free to withdraw at any time without giving a reason.)

Please write your answer here:

Thank you for completing this survey!
03-03-2016 – 08:49

Submit your survey.
Thank you for completing this survey.
