# Supplementary material for: A Web-Based Training Resource for Therapists to Deliver an Evidence-Based Exercise Program for Rheumatoid Arthritis of the Hand (iSARAH): Design, Development, and Usability Testing
Source: J Med Internet Res. 2017 Dec 13;19(12):e411. doi: 10.2196/jmir.8424 (PMC5745347; doi:10.2196/jmir.8424)
Supplement: Multimedia Appendix 3 [file jmir_v19i12e411_app3.pptx]

## Slide 1
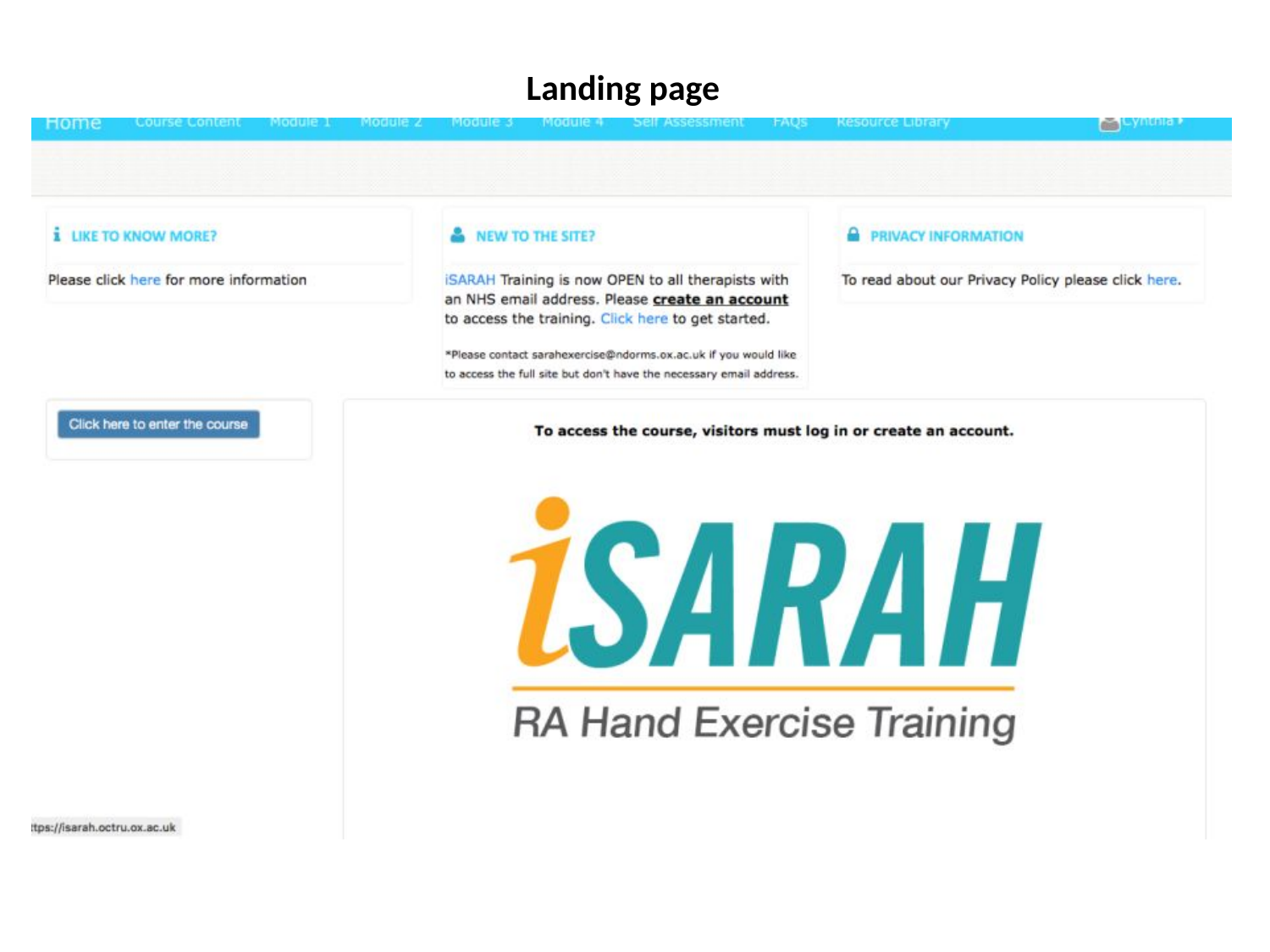

Landing page
#

## Slide 2
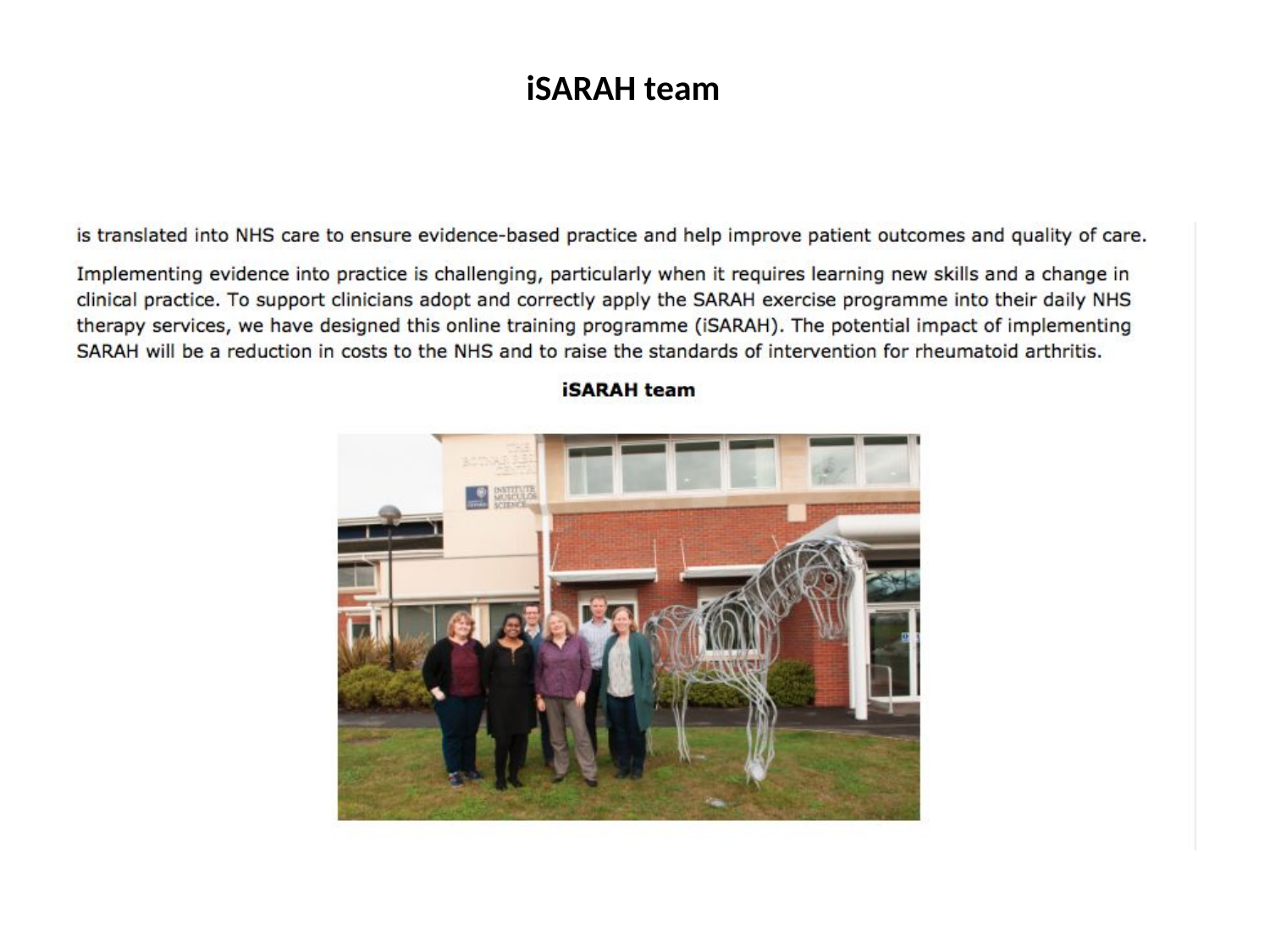

iSARAH team

## Slide 3
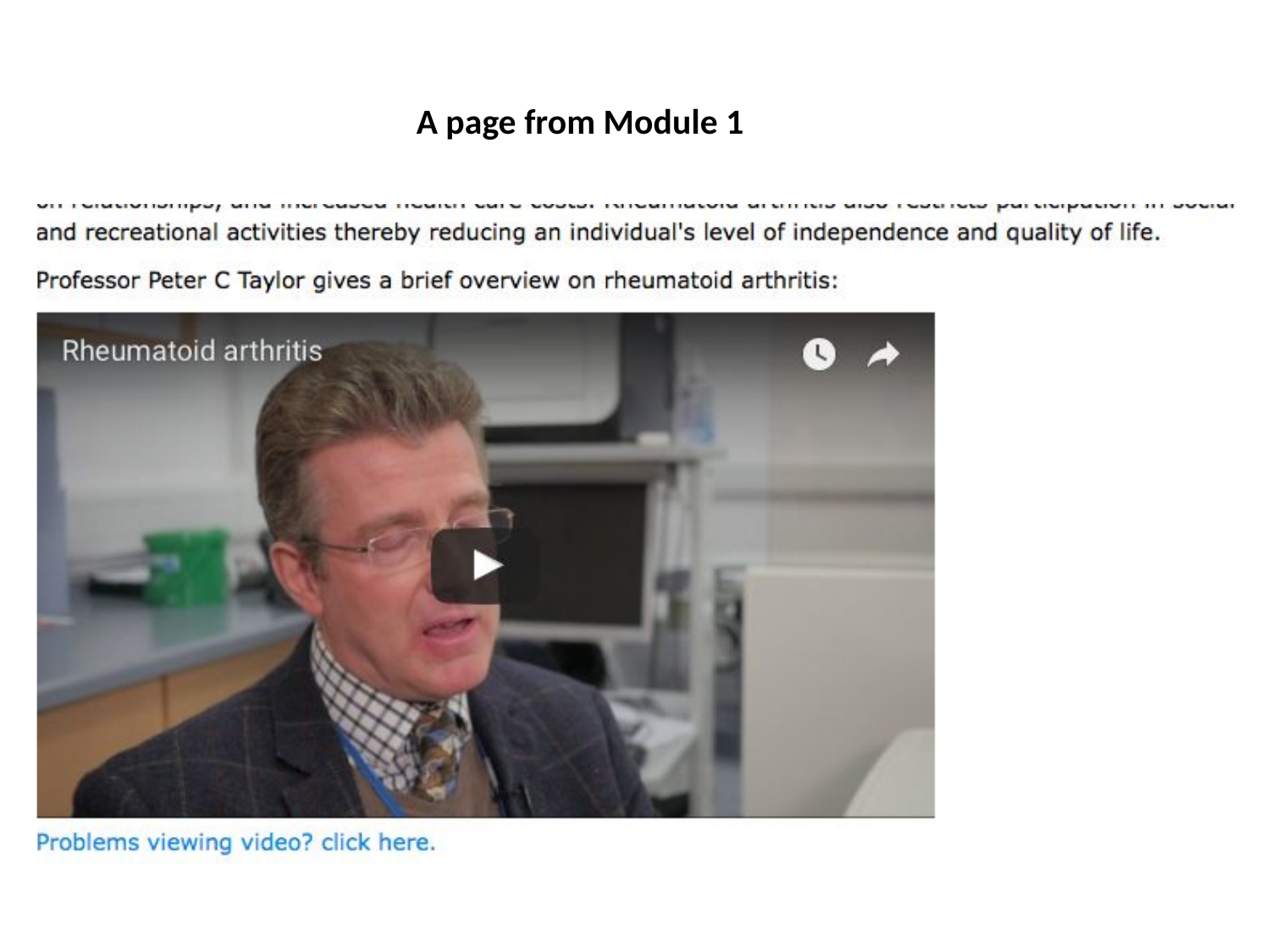

A page from Module 1

## Slide 4
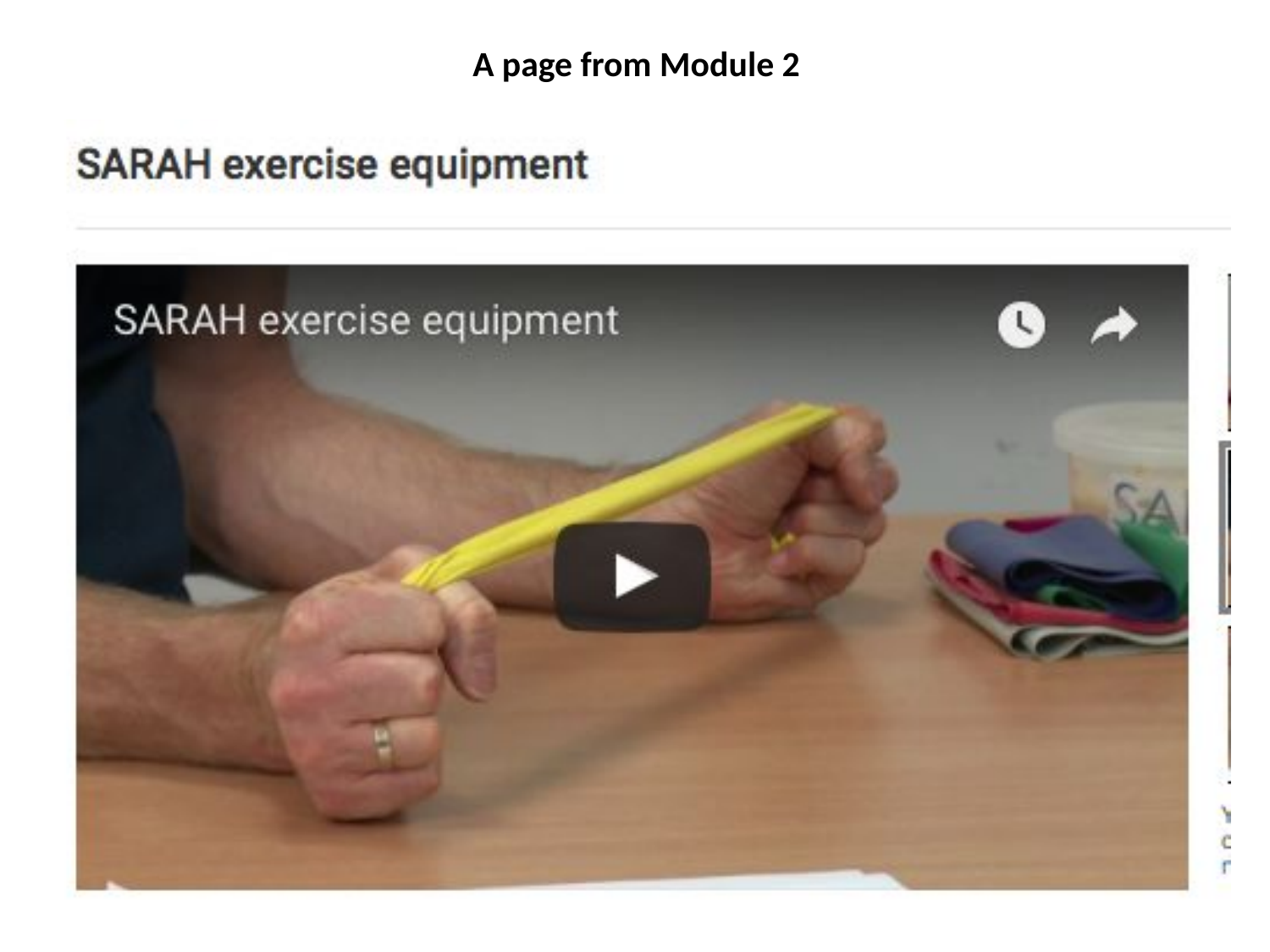

A page from Module 2

## Slide 5
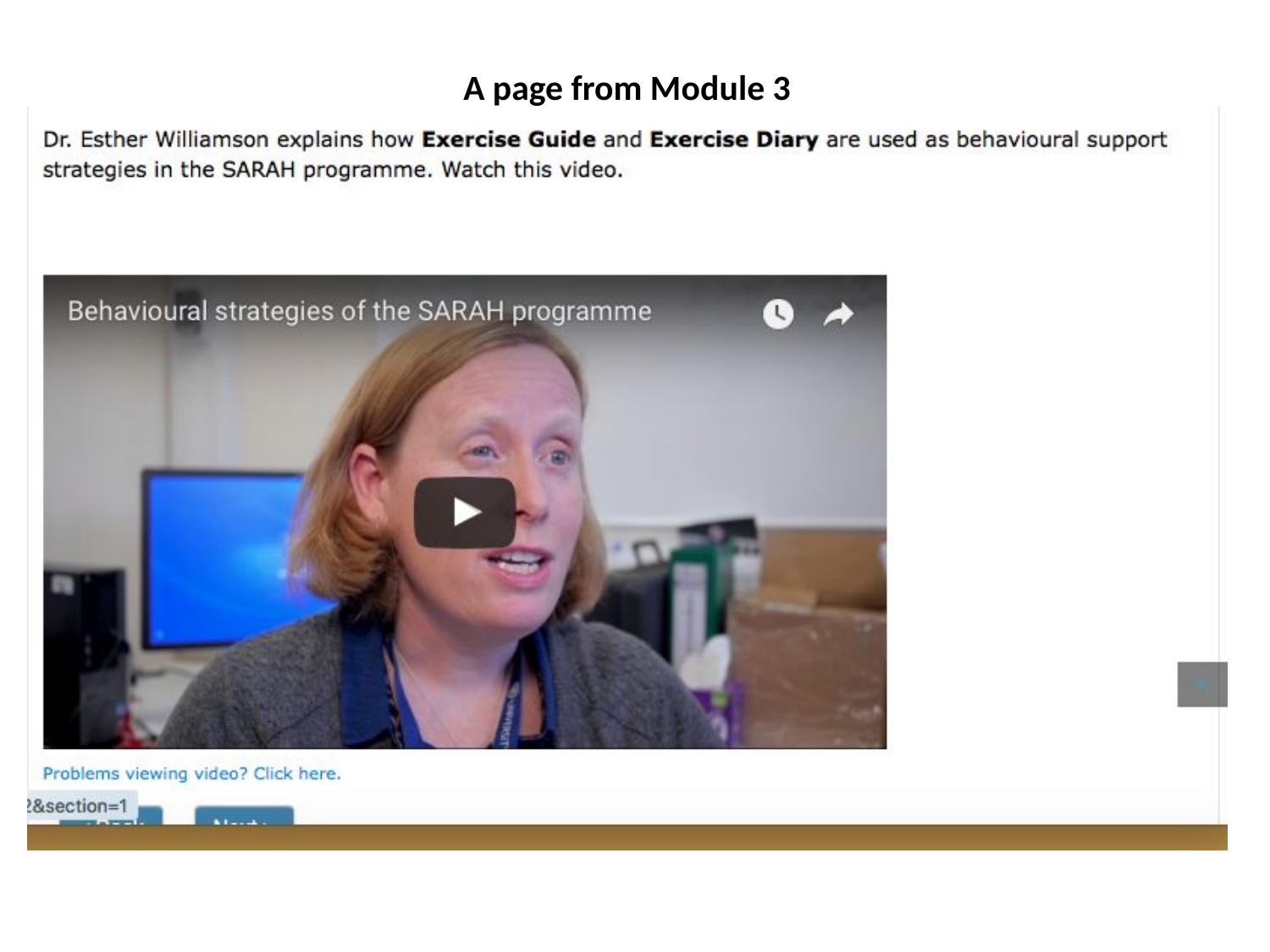

A page from Module 3

## Slide 6
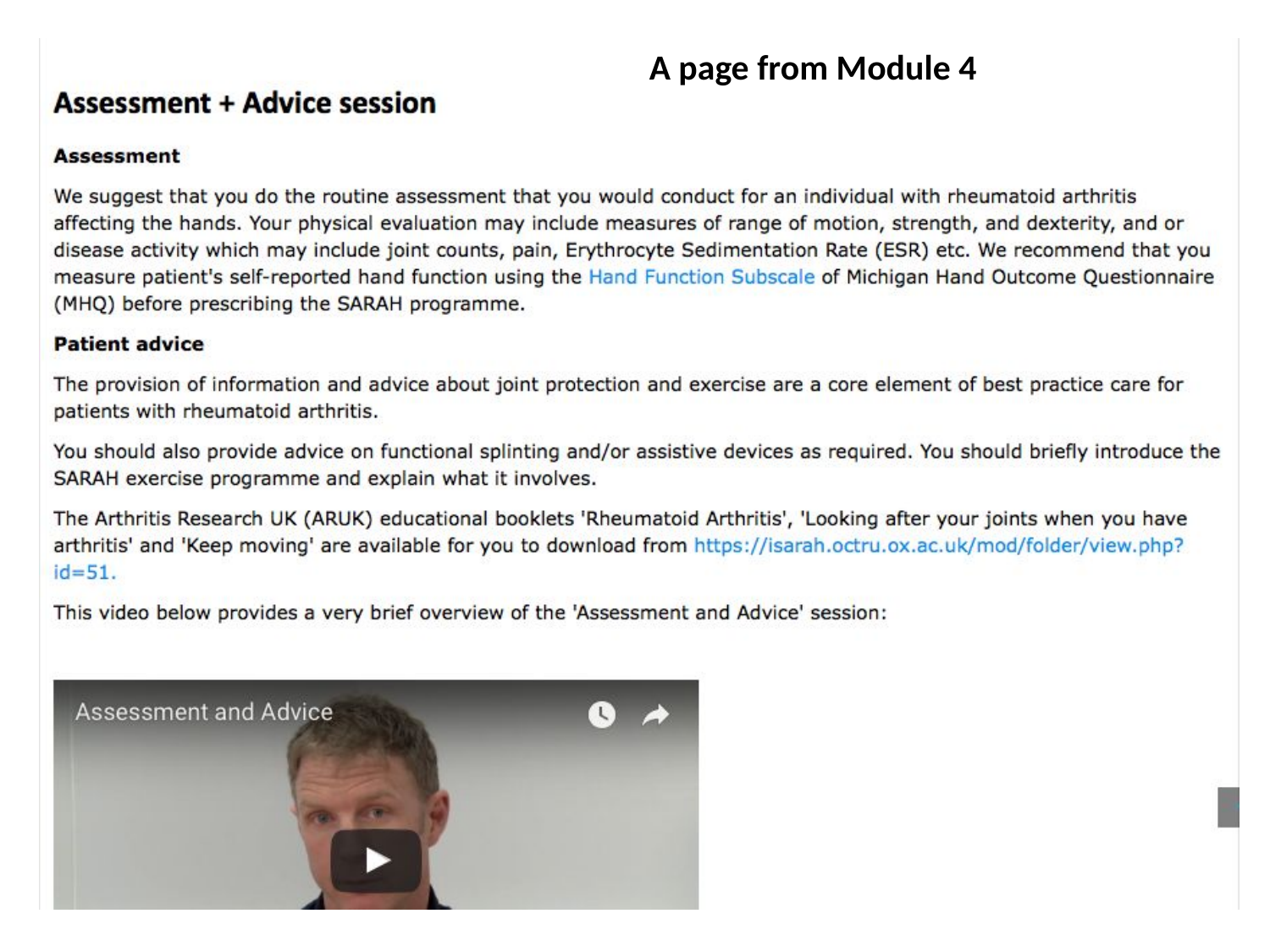

#
A page from Module 4

## Slide 7
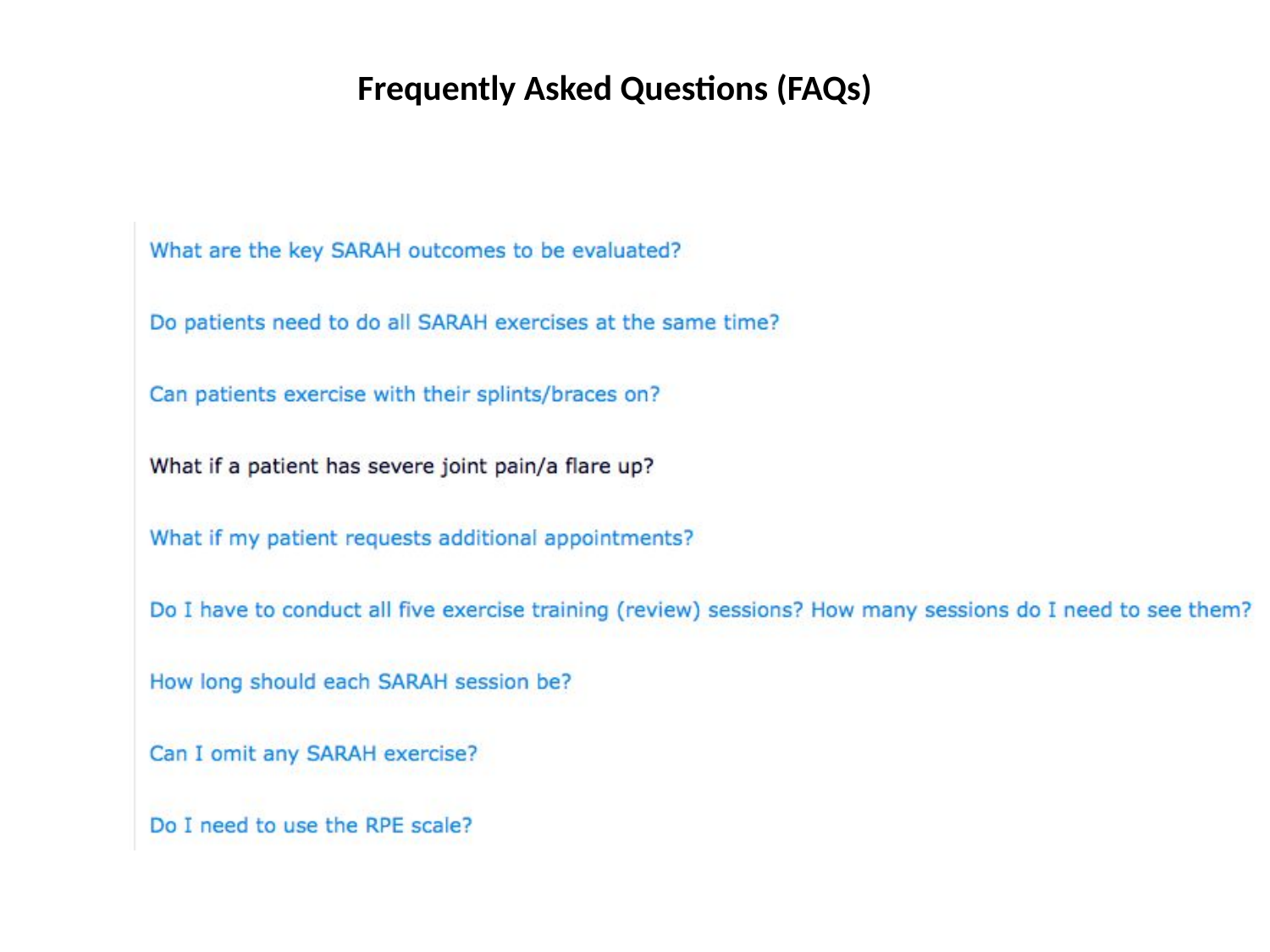

Frequently Asked Questions (FAQs)

## Slide 8
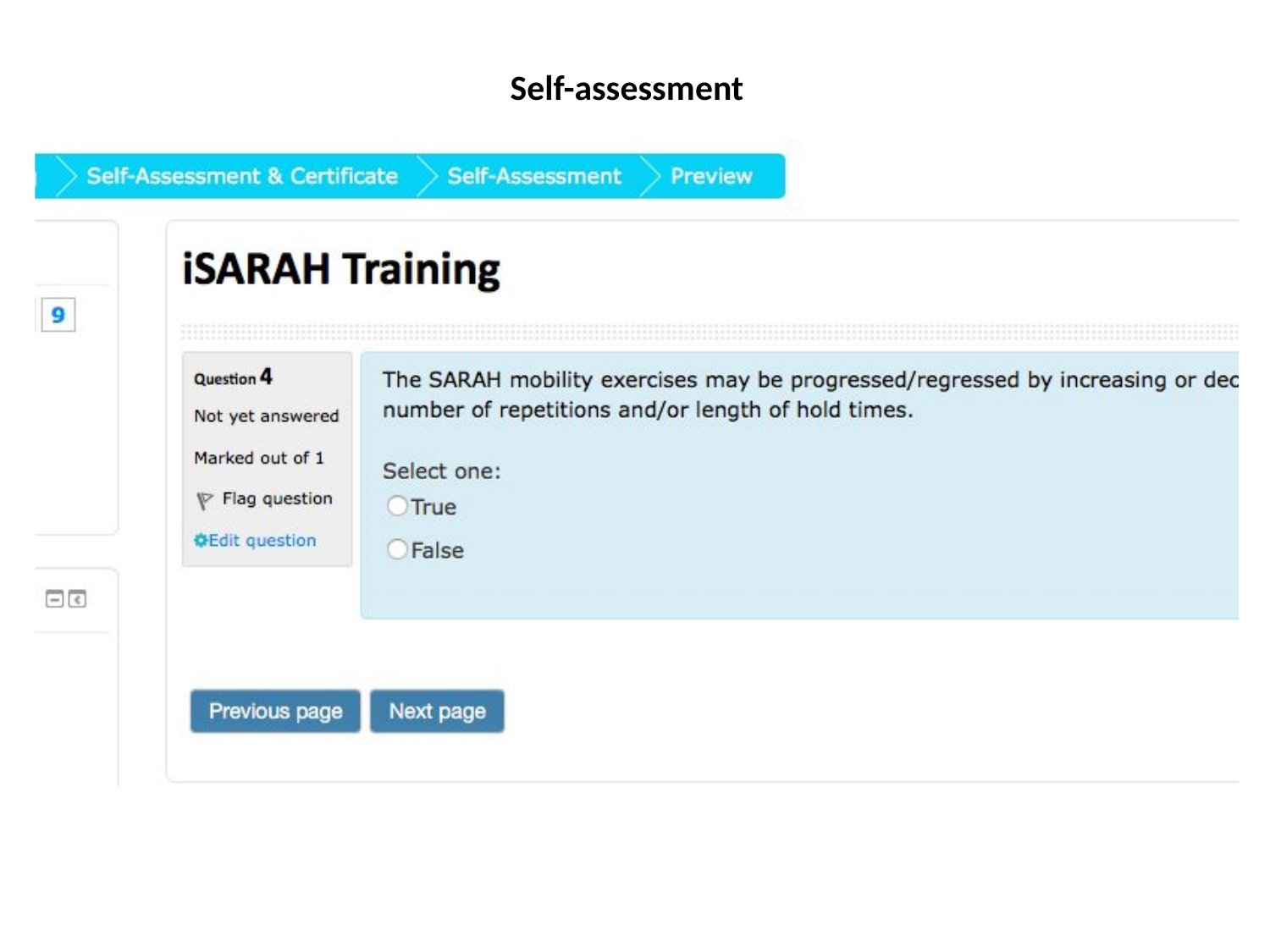

Self-assessment

## Slide 9
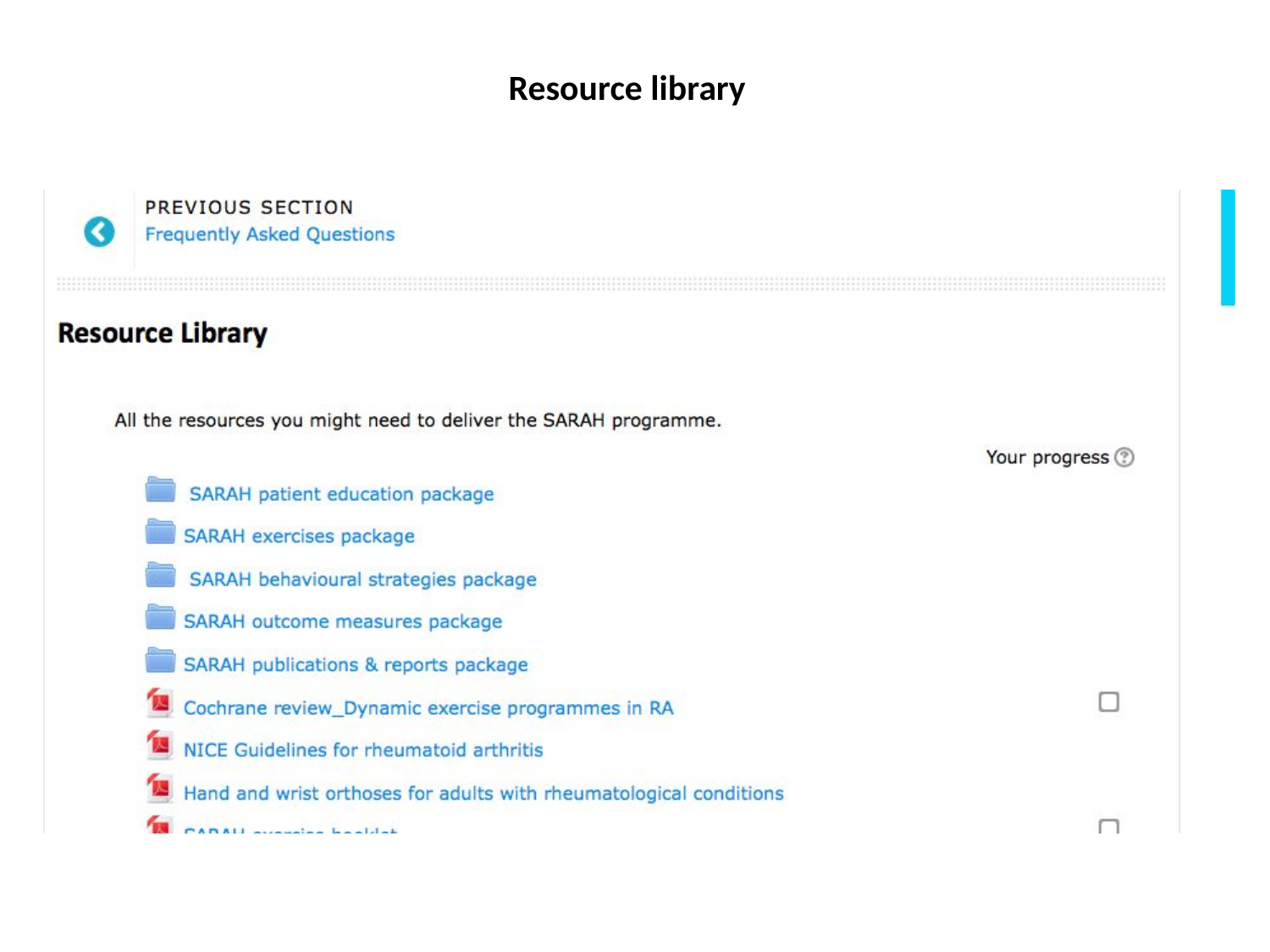

Resource library

## Slide 10
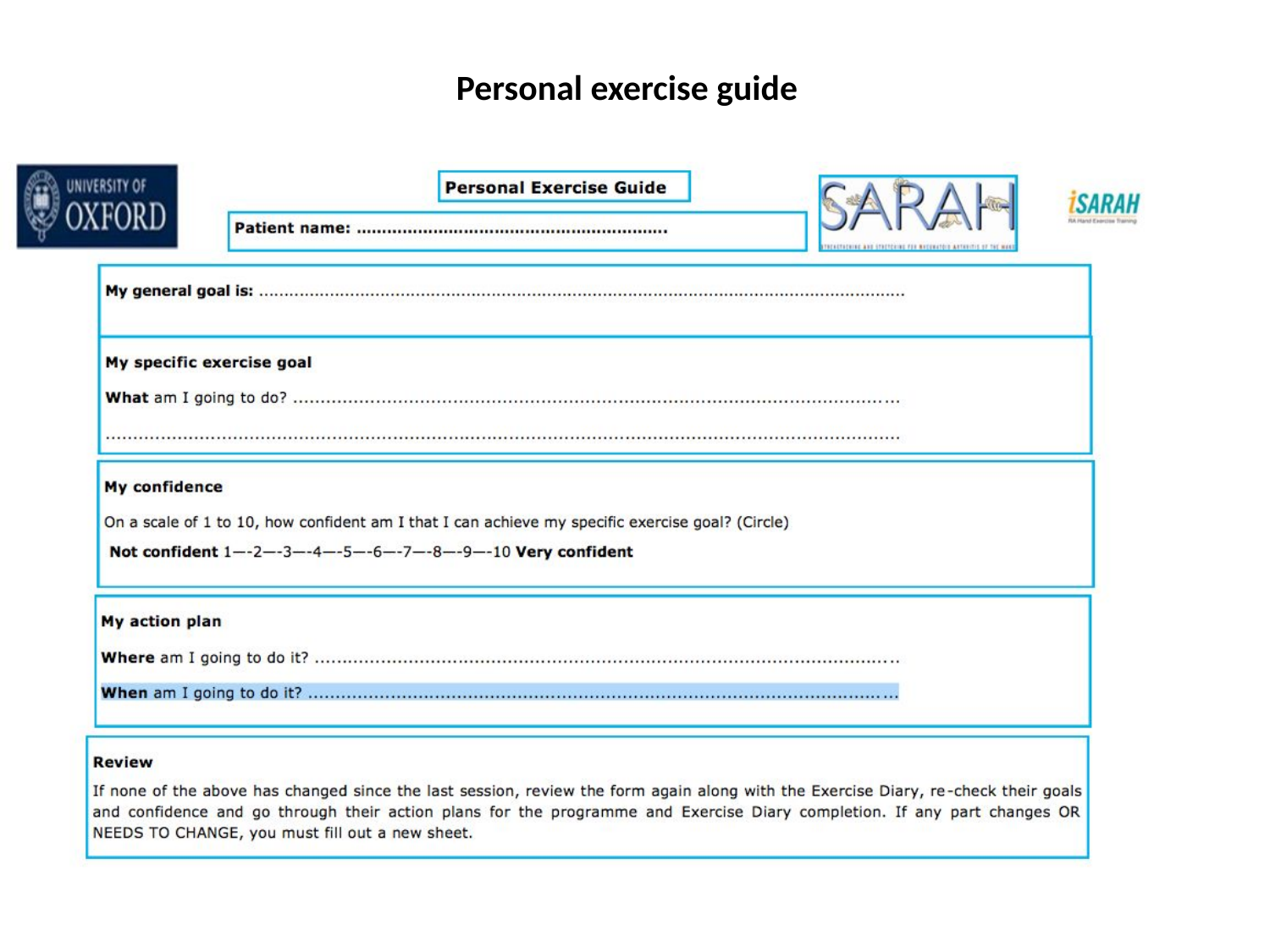

Personal exercise guide

## Slide 11
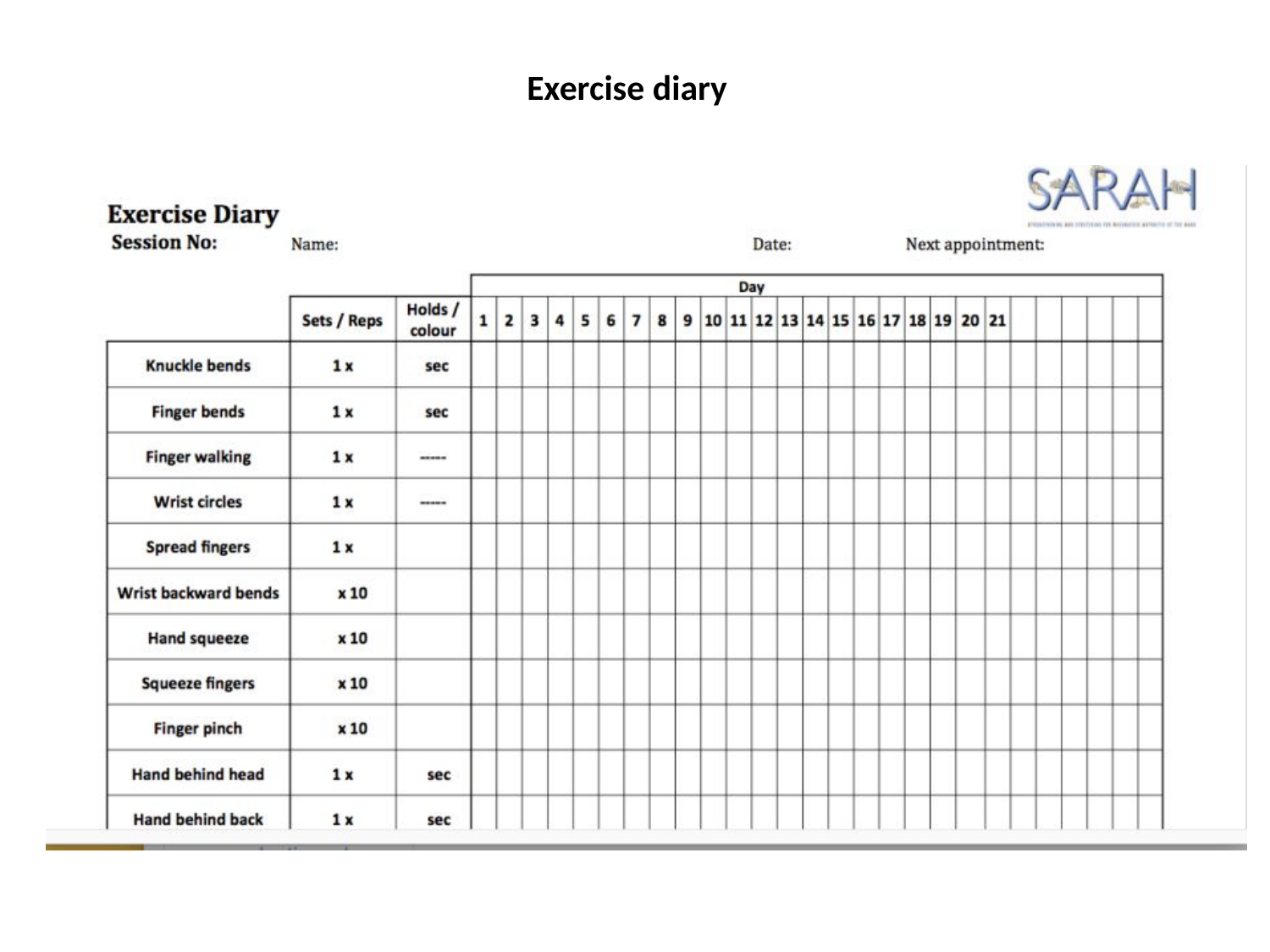

Exercise diary

## Slide 12
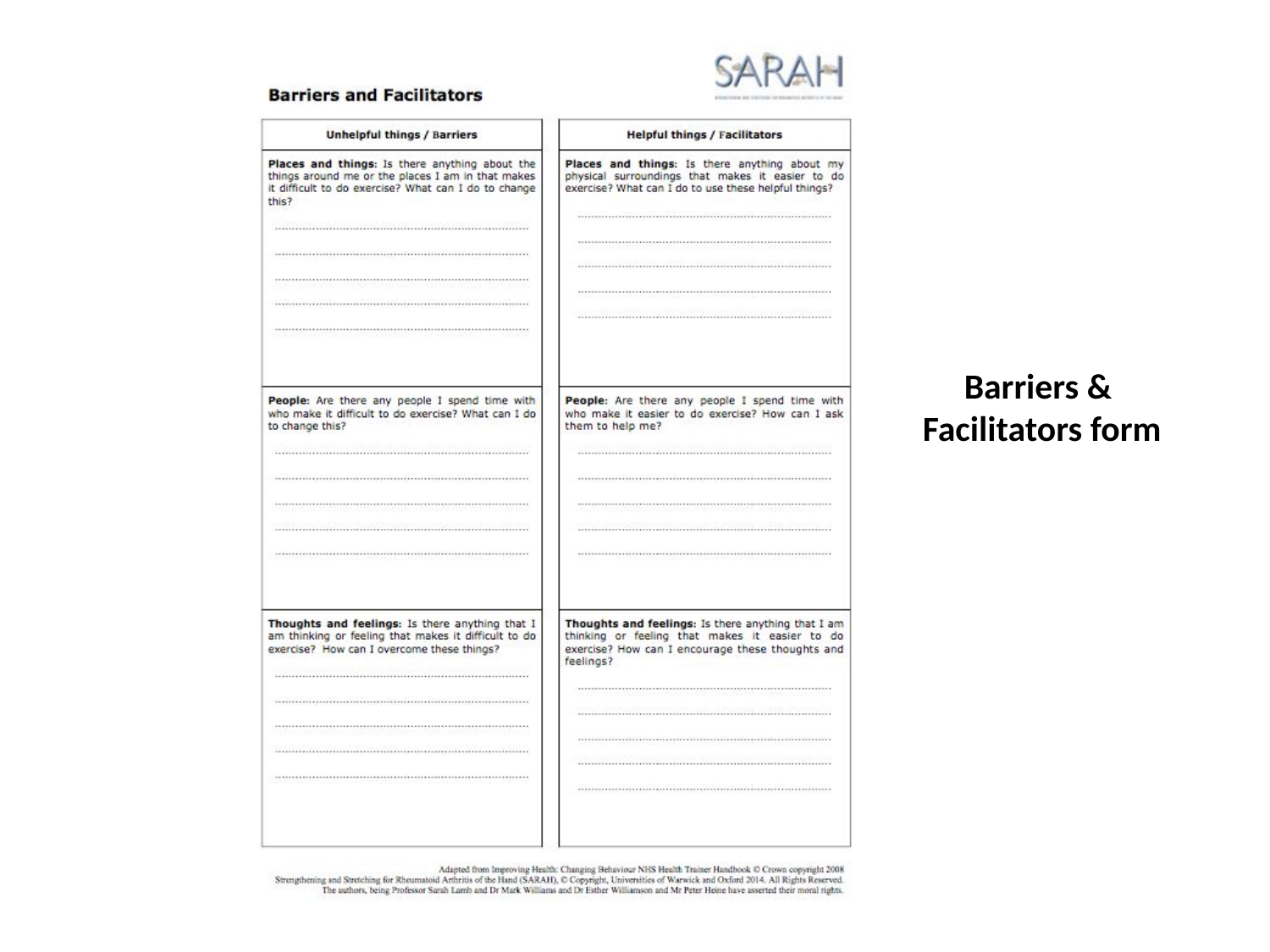

Barriers &
Facilitators form
